# Revolutionizing ocular cancer management: a narrative review on exploring the potential role of ChatGPT

**DOI:** 10.3389/fpubh.2023.1338215

**Published:** 2023-12-15

**Authors:** Saud S. Alotaibi, Amna Rehman, Muhammad Hasnain

**Affiliations:** ^1^Information Systems Department, Umm Al-Qura University, Makkah, Saudi Arabia; ^2^Department of Computer Science, Lahore Leads University, Lahore, Pakistan

**Keywords:** eye cancer, ChatGPT, challenges, oncology, artificial intelligence

## Abstract

This paper pioneers the exploration of ocular cancer, and its management with the help of Artificial Intelligence (AI) technology. Existing literature presents a significant increase in new eye cancer cases in 2023, experiencing a higher incidence rate. Extensive research was conducted using online databases such as PubMed, ACM Digital Library, ScienceDirect, and Springer. To conduct this review, Preferred Reporting Items for Systematic Reviews and Meta-Analysis (PRISMA) guidelines are used. Of the collected 62 studies, only 20 documents met the inclusion criteria. The review study identifies seven ocular cancer types. Important challenges associated with ocular cancer are highlighted, including limited awareness about eye cancer, restricted healthcare access, financial barriers, and insufficient infrastructure support. Financial barriers is one of the widely examined ocular cancer challenges in the literature. The potential role and limitations of ChatGPT are discussed, emphasizing its usefulness in providing general information to physicians, noting its inability to deliver up-to-date information. The paper concludes by presenting the potential future applications of ChatGPT to advance research on ocular cancer globally.

## 1 Introduction

Ocular cancer is not only an issue for the population in developing countries, as developed countries have also reported cases of eye cancer. According to the American Society of Clinical Oncology, more than 3,400 new cases of eye cancer are expected to be diagnosed in 2023 (1). However, in developing countries such as Pakistan, this number is significantly higher. Based on statistics provided by Al-Shifa Trust Eye Hospital, out of the 150,000 individuals diagnosed with cancer each year, 2,200 of them have ocular cancer, resulting in the unfortunate loss of 220 lives annually (2023) (2). It is important to note that there are likely many unreported cases from remote or rural areas. Currently, there is scarce literature addressing ocular cancer, making us pioneers in exploring this issue. Recent work states that cancer is rising worldwide but cancer prevalence data is limited. Several factors contribute to the causes of ocular cancer (3). In order to accomplish this task, we are seeking support from emerging applications of artificial intelligence (AI) and relevant literature on the occurrence of ocular cancer in developing countries. Cancer screening programs have been launched that led to the improvement in survival of patients. However, patient selection and risk stratification are real challenges for caretakers. Moreover, work force and recently prevailing COVID-19 pandemic situation have increased the concerns (4).

The role of AI has been recognized in diagnosis and clinical management of certain cancer diseases (5). AI is excelled at performing well-organized tasks such as image recognition, brain tumor and skin cancer detection (6). AI is playing a clear role in giving support to doctors in their workplaces (7). Since last five years, AI has shown promising role in prospective and clinical trials (8). Open AI application, ChatGPT has shown better performance to examine the European Board of Ophthalmology test, education and knowledge assessment (9).

Emerging literature on ChatGPT and its applications in several fields have motivated us to present an overview of ocular cancer from the literature, shedding light on the challenges faced by common people worldwide. To the best of our knowledge, there currently exists a significant gap in the literature, as no comprehensive studies have yet focused on providing an overview of ocular cancer within the context of generative AI technology. Before this study, a previous review was conducted on the modern treatment of retinoblastoma and its treatment algorithms (10). However, the review focused on data up until 2020, and numerous research studies have been published since then. Another review on the topic of “ocular cancer” specifically addressed common ocular tumors, as well as their diagnosis and treatment technique (11). The most recent review article focuses on the role of deep learning models in cancer care. Deep learning significantly reduces costs in baseline imaging. Ethical issues related to the use of deep learning models were also studied in the review article (12). However, these review articles did not discuss the role of generative AI models and their applications in diagnosing ocular cancer. In the field of oncology and particular ocular cancer, the specific role and impact of ChatGPT application remains relatively unexplored. ChatGPT offers to address challenges related to ocular cancer by assisting in early detection using data analysis and offers tailored treatment recommendations.

To understand the versatility and widespread applicability of AI in healthcare, particularly in the context of ocular cancer, is an interesting topic to explore. The promising use of ChatGPT applications in ophthalmology for question-answering has been previously outlined, assessing the enhanced performance of large language models in healthcare (13). Additionally, a recent research highlights the application of ChatGPT in diagnosing intraocular tumors and ophthalmic pathologies (14). Moreover, the improved competency of generative AI applications in diagnosing diverse ocular conditions has further underscored its potential in the field of ophthalmology (15).

To conduct a narrative review on the role of ChatGPT application in ocular cancer is necessary and timely. Earlier mentioned studies highlight the rapid advancement of generative AI applications in healthcare, understanding the ChatGPT’s capabilities in diagnosing and treating the ocular cancer becomes crucial. Insights drawn from existing literature can complement the existing practices and contribute to the management of ocular cancer diagnosis and treatment. Therefore, the main aim of this study is to bridge the gap in the literature by evaluating the specific contributions of AI applications, like ChatGPT, in diagnosing and treating ocular cancer. This includes identifying challenges faced by patients and examining how ChatGPT can help overcome these obstacles. Additionally, our review seeks to consolidate emerging information on the potential benefits of integrating ChatGPT in oncology. Furthermore, this narrative review outlines significant implications for future research in this rapidly evolving field.

Overall this narrative review contributes to the literature as follows:

- This paper presents an overview of the important types of ocular cancer based on literature and resources related to ChatGPT applications.
- This paper highlights the potential role of ChatGPT applications in improving the health outcomes of individuals with the ocular conditions.
- This paper identifies several challenges of ocular cancer and suggests ways to overcome them.
- This paper presents some important research implications for future studies in the area of AI applications and their use in the context of ocular cancer.

Section 2 outlines the methodologies employed in this paper. In Section 3, we provide the profound results and engage in in-depth discussions concerning the crucial role and applications of ChatGPT in addressing ocular cancer. Lastly, Section 4 succinctly summarizes the cornerstone findings of this research, thereby underscoring its significance.

## 2 Methods

Methodological approach adopted in this study is based on the narrative review that presents a qualitative interpretation of publications aimed at discussing the literature on the issues that may increase the debate from scientific community (16).

### 2.1 Search strategy and selection criteria

To conduct this review, relevant references were identified through the comprehensive searches in PubMed, Springer, ACM Digital Library and ScienceDirect databases. The search terms utilized for literature retrieval encompassed “Ocular cancer” OR “Eye cancer,” in combination with “ChatGPT application” OR “Open AI application”.

Table 1 illustrates the strategic employment of search keywords across digital libraries.

**Table 1:** Search keywords used on digital libraries.

Search keyword	Digital library	URL
(((Ocular Cancer) AND (ChatGPT)) OR (Eye Cancer)) AND (ChatGPT)	PubMed	https://pubmed.ncbi.nlm.nih.gov/37266720/
(ocular AND cancer AND ChatGPT AND application)	Springer	https://link.springer.com/advanced-search
(Ocular cancer or eye cancer And ChatGPT application)	ScienceDirect	https://www.sciencedirect.com/search?qs=Ocular%20cancer%20or%20eye%20cancer%20And%20ChatGPT%20application

Data extraction was performed by authors in three steps as follows:

1. All articles covering the topic were selected for the final inclusion in the review.
2. Authors read the articles and summarized the main features such as ocular cancer types, causes, treatment, future directions and references.
3. Authors read the articles and removed the overlapping and redundancy among titles.

**Figure 1:**
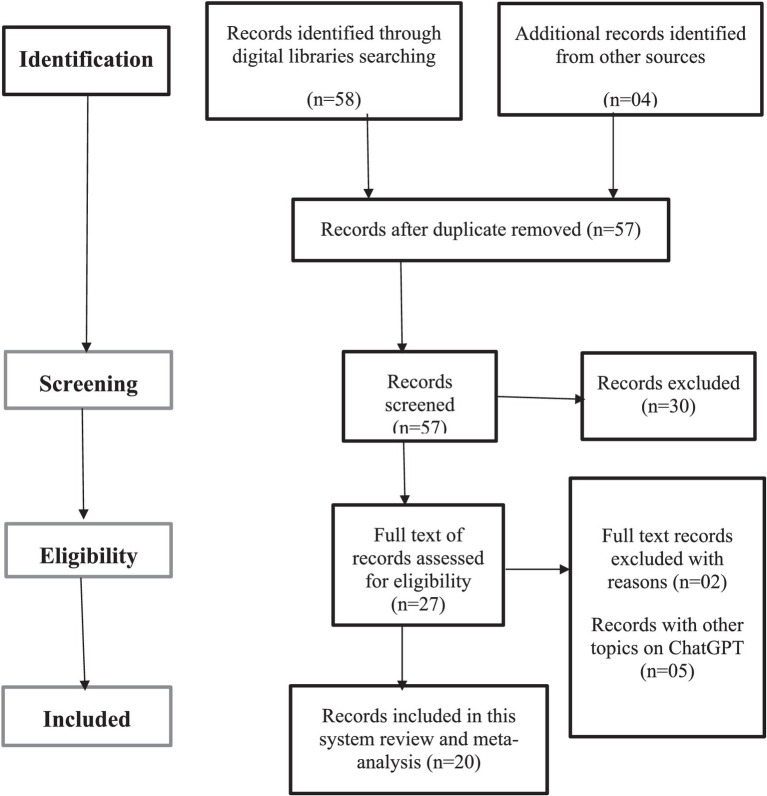
PRISMA flowchart.

Authors (SA, AR, and MH) contributed to the data extraction from the full-length articles. The search scope of this study was limited to the AI application and ocular cancer. Other articles were manually searched and added to the review to provide additional knowledge on the topic. Overall, 20 articles were selected for the inclusion in this review (Figure 1). For this purpose, we followed the PRISMA guidelines (17).

### 2.2 Inclusion and exclusion criteria

Studies’ inclusion and exclusion criteria are given as follows:

Full-text research articles published in English language were considered in this narrative review. Specifically, articles published within the last 4 years (from 2020 to 2023) were eligible for inclusion. To ensure the integrity of the review, duplicate articles were excluded. Additionally, grey literature, and book chapters were omitted from this review study. Moreover, research articles that did not relate to ocular cancer and ChatGPT were also excluded.

### 2.3 Document screening and data extraction

Titles and abstracts of studies were checked for de-duplication. Researchers of this study independently reviewed each document against the eligibility criteria. Following the initial screening, full-text records were collected to confirm the eligibility. No disagreement was observed between reviewers.

## 3 Results and discussion

Based on the search strategy in four databases, 62 records were collected (Figure 1). After removal of duplicate records, 57 articles were further screened for eligibility. Of the remaining records, 30 were excluded using the exclusion criteria. Resulting 27 records were evaluated based on the full-length articles, after that 7 records were excluded. A total of 20 articles were selected in this narrative review. Subsequently results have been discussed in the following.

### 3.1 Important types of ocular cancer

To investigate the strength of ChatGPT in the identification of ocular cancer types, we present the comparison of literature and ChatGPT-generated text on ocular cancer types. Prominent venues published articles on various types of ocular cancer. They reported Retinoblastoma and Ocular Surface Squamous Neoplasia (OSSN) in the study and found that the former type was more common than the latter one (18). In the following table, we outline ocular cancer types, their respective causes, treatments, future directions and relevant references.

The data presented in Table 2 varies due to differences in treatment oprions, location, stage and individual patient characterization. Seven types of ocular cancer have been identified from the ChatGPT responses. UV exposure is mainly pointed out as the main cause of different ocular cancer types. The table outlines future directions, offering potential areas where ongoing research projects could be efficiently implemented. Aside from the causes of ocular cancer, literature reports suggest that genetic mutation, ultraviolet radiation, age, environmental exposures, viral infections and hereditary conditions may also contribute to eye diseases (29, 30). Notably, Ocular Adnexal Malignancies has been missed by the ChatGPT application (31). Regarding treatments as listed in Table 2, the shift towards personalized medicine, especially in conjunctival melanoma and ocular melanoma, reflects a growing trend in oncology, moving away from generalized treatments towards more individualized techniques. However, the treatment for Choroidal Metastasis has not been widely explored in the literature. This gap is exemplified by one of the studies by Au (28), as listed in Table 2, which is marked with a ‘Did not reveal’ status. This indicates a significant omission of a treatment strategy in the study. Furthermore, the potential role of ChatGPT in enhancing healthcare experiences, particularly for choroidal metastasis, signals an increasing recognition of AI’s role in healthcare. However, this appears as an isolated mention, indicating an early stage of AI integration in this field.

**Table 2:** Summary of ocular cancer types.

Ocular cancer	Causes	Treatment	Future directions	References
Retinoblastoma	RB1 gene mutations	Chemotherapy, radiation, and surgery	Targeted therapies, gene therapies, and immunotherapies	Dimaras et al. (19) and Ancona-Lezama et al. (10)
Ocular melanoma	Genetic mutations, UV exposure	Surgery, radiation, and immunotherapy	Personalized medicine, targeted therapies, and immunotherapies	van Poppelen et al. (20) and Ruan et al. (21)
Conjunctival melanoma	UV exposure, genetic mutations	Surgery, radiation, and immunotherapy	Targeted therapies, and immune checkpoint inhibitors	Brouwer et al. (22)
Ocular Surface Squamous Neoplasia (OSSN)	HPV infection, UV exposure	Surgery, topical chemotherapy, and cryotherapy	HPV vaccination, targeted therapies, and immunotherapies	Hӧllhumer et al. (23) and Kozma et al. (24)
Intraocular lymphoma	Unknown (possibly viral infections)	Chemotherapy, and radiation	Immunotherapies, targeted therapies, and understanding viral links	Ghesquieres et al. (25) and Hearne et al. (26)
Lacrimal gland tumors	Unknown (genetic and environmental)	Surgery, and radiation	Improved understanding of tumor biology, and targeted therapies	Emerick et al. (27)
Choroidal metastasis	Breast and lung cancers	Did not reveal	ChatGPT may help enhance human experience in healthcare	Au (28)

A detailed analysis of the data in the Table 2 reveals that while ChatGPT is a valuable resource, it is not without limitations as a source of information for health data, particularly in the context of ocular cancer types. Several important aspects of ocular cancer have been overlooked by the ChatGPT application. This discrepancy may stem from the data used to train ChatGPT, which remains current only until 2021. The recent advancements in ocular cancer research have not been incorporated into the training of the OpenAI application. Consequently, we are unable to access updated and highly accurate information regarding the causes and treatment of ocular cancer.

### 3.2 Ocular cancer challenges

This section presents some challenges associated with the ocular cancer.

#### 3.2.1 Limited awareness

In many developing countries, a critical lack of awareness about the signs and symptoms of eye cancer persists, often leading to delayed diagnosis and treatment. This issue is particularly true in rural areas where access to healthcare and health education is frequently limited. Targeted public health campaigns and educational initiatives are essential to increase awareness among the general population, healthcare professionals, and policymakers. Research has shown that individuals in resource-limited economies often have limited awareness and knowledge about the ocular cancer (32). Therefore, education is a key factor in elevating public awareness about eye cancer, especially in these vulnerable populations.

#### 3.2.2 Limited access to health care services

The availability of specialized ophthalmic oncology services, including expert ophthalmologists and oncologists, can be markedly limited in certain regions of the world. This scarcity significantly hinders the ability of people with eye cancer to receive timely and appropriate care. A study conducted in the Middle East, West Asia, and North African regions investigated the treatment capabilities concerning retinoblastoma. This study further analyzed resources such as diagnostics, advanced treatment, focal therapy and chemotherapy (33). A significant disparity in the availability of resources among these countries was evident. Consistent with previous studies, recent research also showed that Retinoblastoma care facilities were notably limited in Ethopia, predominantly restricted to urban areas (34).

#### 3.2.3 Financial barriers

The expense of diagnostic procedures, surgical procedures, radiation therapy, and post-operative care may be prohibitive for patients due to the high cost of eye cancer treatment. Cost is one of the leading factors that increases difficulties in receiving better treatment modalities (35). The literature overlooks this issue associated with the high cost of ocular cancer treatment. One study solely presents the use of convolutional neural networks for detection of ocular abnormalities (36). Similarly, another study examines the ocular melanoma in Polish population and does not present cost associated with this type of ocular cancer (37).

Government initiatives, insurance coverage, and support programmers are just a few examples of accessible and cheap healthcare choices that can assist ease the financial strain on impacted people and their families. There is a high cost on travelling and referrals for the treatment of eye cancer (38). Delays in care may cause the serious concerns and challenges in receiving the definitive treatment.

#### 3.2.4 Infrastructure and resources

Effective management of eye cancer depends on the accessibility of cutting-edge medical infrastructure, including diagnostic facilities, treatment tools, and skilled healthcare personnel. Diagnosis, therapy, and overall patient outcomes can all be improved by enhancing the infrastructure and resources devoted to eye cancer care.

Several other challenges contribute to the rise of eye cancer in different settings. United Kingdome healthcare system was impacted by the COVID-19 pandemic. There was a considerable decrease in melanoma referral patterns in UK during the first wave of COVID-19 in 2020 (39). Since then recovery plan has been initiated by the ocular oncology services.

**Figure 2:**
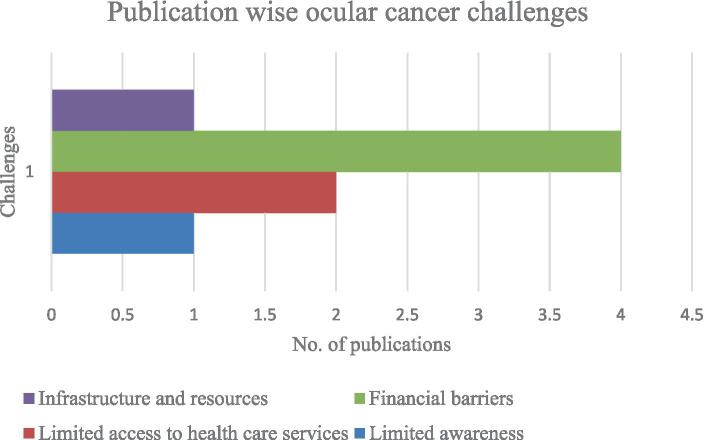
Publication wise ocular cancer challenges.

As seen in Figure 2, financial barriers have been widely studied as ocular cancer challenges in the literature. The second most examined ocular cancer challenge is limited access to healthcare services, followed by infrastructure and resource constraints, and limited awareness, each documented in a single publication.

### 3.3 Potential role of ChatGPT to overcome ocular cancer challenges

ChatGPT is designed to converse with users in natural language, responding to their inquiries and assertions in a manner that simulates online discussion (40). Capable for addressing various topics, from general knowledge questions to more specialized inquiries spanning healthcare, travel, technology, and many other topics, the Chatbot can comprehend and react to a wide range of topics.

Here are some potential ways that GPT-based chatbots could be used to overcome ocular cancer challenges:

- **Education and awareness:** patients can get information from chatbots about ocular cancer symptoms, risk factors, and treatment options. Cancer patients continuously require online sources to educate themselves. ChatGPT can provide them information about ocular cancer. This may aid in raising awareness and promoting early detection of ocular cancer, both of which are essential for effective treatment outcomes. Recent work evaluate the role of ChatGPT in providing recommendations about breast, lungs, and prostate cancers (41). However, this study overlooks the role of ChatGPT in giving information to patients on ocular cancer.
- **Support for patients and caregivers:** ChatGPT might offer help and direction to patients and caregivers by responding to their frequent inquiries and sharing details about available resources and support services. ChatGPT helps patients cope with the emotional impact of cancer, while patient caregivers assist them in managing the distress caused by the disease. ChatGPT reassures patients and caregivers that they are not alone (28). It offers valuable social support to patients in their personal lives.
- **Telemedicine and remote consultations:** ChatGPT might make it easier for patients and medical professionals to interact and communicate online during telemedicine and remote consultations. Patients who reside in distant or underdeveloped locations, where access to specialized healthcare services may be constrained, may find this to be of great use.
- **Research and development:** ChatGPT could support ocular cancer research and development by enabling access to information and insights that could guide the creation of novel therapies and treatments.

In developing countries, dealing with the effects of ocular cancer requires a comprehensive approach. This entails disseminating knowledge about the condition, boosting patient and family support networks, expanding access to specialized care, and promoting early identification through routine eye exams. Additionally, putting regulations in place to reduce the cost of ocular cancer treatments will aid in more effective management and treatment for affected individuals. Beyond these ChatGPT applications, existing literature also emphasizes the potential applications of ChatGPT in ocular cancer. Table 3 presents a summary of the literature on ocular cancer along with its ChatGPT applications.

**Table 3:** Potential role of ChatGPT application.

Problem	Methods	Advantages	Disadvantages	Recommendation	References
Operative notes documenting	ChatGPT application	Supports to write operative notes	Sufficient in fully comprehending the requirements	ChatGPT can be employed with human expertise.	Waisberg et al. (42)
Inherent complexity of operative notes	ChatGPT application	Plays promising role in healthcare	Certain privacy concerns are overlooked	Crucial consideration to overcoming the data privacy of patients	Lawson McLean (43)
Answering question related with ophthalmology	ChatGPT application	Provides 50% right answers to questions	Accuracy is not up to the required standard	Updated information can be used to train ChatGPT and receive more accurate results	Mihalache et al. (44)
Accuracy in answering the ocular symptoms	ChatGPT application	Shows better accuracy ChatGPT-3.5 (59.5%) and ChatGPT-4 (89.2%)	ChatGPT exhibits weaknesses in its medical acumen compared to human experts.	Continuous improvement in ChatGPT could minimize the inaccuracy in its performance	Pushpanathan et al. (45)

The current literature on cancer and related topics regarding ChatGPT applications is rapidly emerging. Numerous published works have highlighted the role of ChatGPT applications in healthcare, with a particular focus on cancer (46, 47). However, only a limited number of articles have been published on the potential role of ChatGPT applications in enhancing the health conditions of ocular patients. These studies primarily concentrated on generating operative notes during surgical procedures (42, 43). This demonstrates a clear trend towards leveraging the ChatGPT application to enhance efficiency, as highlighted by its use in answering specialized questions and documenting operative notes. While ChatGPT is emerging as a useful tool in oncology, its effectiveness is tempered by concerns over accuracy and comprehension. For instance, ChatGPT achieves only 50% accuracy in some cases (44), and shows limitations in understanding complex medical treatments. In addition, Data privacy has not been adequately addressed in the process of generating these operative notes (44, 45). The data utilized in surgical operations is highly sensitive and must be shared with the surgical staff in a cautious and meticulous manner.

ChatGPT application demonstrates its widespread use in many areas. GPT’s role in identifying the rare eye disease has been explored in a research study (48). ChatGPT works as a consultation assistant for patients and family physicians for referral suggestions. In addition, ChatGPT application helps junior ophthalmologist in diagnosing the rare eye diseases. All ChatGPT users need to be careful and cautious to acknowledge the adequate referrals and verifications in clinical settings. ChatGPT application is being applied in the world of intelligent diagnostics, Literature reveals that ChatGPT has shown potential in radiology and interpretation of the clinical images (49). It has remarkably improved the clinical workflow and responsible utilization of radiology services. Higher accuracy in clinical decision making has been seen using the ChatGPT application (50). Trusting the ChatGPT application is crucial for its adoption in healthcare, considering that ChatGPT wasn’t primarily developed for healthcare purposes. Overreliance on this technology might potentially result in disseminating false information and health risks (51). Therefore, our efforts should focus on further enhancing the ChatGPT application to differentiate between queries that it can manage effectively and those that should be redirected to human experts.

Overall, these insights indicate a future in which AI could play a supporting and integral role in ocular cancer, necessitating continuous advancements.

### 3.4 Research implications

Currently, AI applications have demonstrated a significant role in ophthalmology, particularly in the areas of imaging and data measurement. Conventional methods to diagnose ophthalmic disease depend on the clinical assessment and image capturing devices for the different modalities (52). These diagnostic methods are time consuming and costly, and make ophthalmology one of the areas suited to the recent deep learning models. ChatGPT application is widely used in training curricula in medicine. Although ChatGPT application is still in the research stage, it has shown promising results, particularly in ocular cancer, where the potential role of AI is extensively explored in the literature (53). In this context, ChatGPT serves as a valuable tool to assist researchers and practitioners in successfully transitioning to AI-based approaches in the field of ocular cancer. By leveraging the capabilities of ChatGPT, professionals can benefit from its insights and assistance, ultimately leading to improved outcomes for patients. By sharing insights and potential data on the AI platform, physicians may promote greater communication among other doctors and patients (54). This way, physicians can make better decisions by accessing the patients’ data and opinions from colleague doctors.

Recent work explores the use of ChatGPT in the simulated “Ophthalmic Knowledge Assessment Program” OKAP examination. ChatGPT achieved an accuracy of 59.4% on the OpthoQuestions testing set compared to a 74% accuracy by human on the Basic and Clinical Science Course (BCSC) test set (13). Another research study reported that ChatGPT attained 46% accuracy in answering OpthoQuestions for the preparation of board examinations (44). Compared to human performance on the testing set, ChatGPT exhibited slightly lower performance (55). However, ChatGPT’s performance is noteworthy and could be further enhanced with the use of updated versions of ChatGPT. The latest versions of ChatGPT can be trained with the most recent oncology data, ensuring higher accuracy and relevance in healthcare. The recent version of ChatGPT (v. 3.0) demonstrated limitations in controlling a question’s difficulty as well as its cognitive level. This limitation should be considered for future versions of ChatGPT applications.

In a most recent study, it was revealed that ChatGPT’s performance in offering recommendations for cancer treatment deviated from NCC guidelines (56). This is a major concern that needs to be addressed in the near future. Although, ChatGPT largely adhered to the NCC guidelines, instances of partial deviation should be addressed in upcoming versions of the ChatGPT application.

ChatGPT could be future of the AI technology due to its features. However, we need to evaluate and monitor it while using for the communication of the eye cancer. Therefore, potential bias must be eliminated to provide equal information to all populations. ChatGPT shows limitations to answer the queries on the updated information (57). ChatGPT is trained on the data collected prior to 2021, and emerging information on eye cancer is not accurate and its accuracy may be increased due to its training on the data collected to date. While ChatGPT version 4.0 has addressed this deficiency to some extent, there is a continued need for consistent enhancements in future versions. A recent study reports that none of the existing studies explored the proposal of AI model to diagnose the Retinoblastoma (58). Common types of Retinoblastoma diagnosis were based on the clinical examination and special signs (59). One of the leading works used AI and Machine learning ML models to analyze the images and numerical data (60). At this stage, the interpretation of results is a very sensitive task in cancer research. Therefore, ChatGPT could be incorporated to make interpretation easier in studies involving the data regarding ocular cancer. Additionally, AI models may be used in the future to detect and refer ocular oncologies, specifically focusing on the lesions where treatment is required.

The emphasis on telemedicine applications extends to ocular cancer. Recent work highlights the considerations for implementing teleophthalmology in Brazil as a future research direction (61). Incorporating ChatGPT application with other technologies may give rise to favourable approaches for physicians and patients in oncology.

A large volume of unstructured information in the form of electronic health records (EHRs) is rather difficult to analyze using traditional techniques (62). ChatGPT has the potential to revolutionize oncology by analyzing vast amounts of information, uncovering trends and patterns that might elude human detection. This innovative tool could enable physicians in oncology to make more precise decisions regarding diagnosis, treatment, and cancer prevention, leading to highly personalized treatment plans tailored to individual patients’ needs. Moreover, the strategic integration of ChatGPT with EHRs could significantly reduce the workload of frontline healthcare workers in oncology, providing crucial support and alleviating workforce shortages, particularly during times of crisis (63). Due to increased concerns about data security and privacy among AI ChatGPT users, it could be integrated with blockchain technology to ensure data consistency and protect medical data, thereby providing traceable and secure usage in oncology medicine (64). Furthermore, for the advancement of medical research in oncology, ChatGPT could become a faster and more reliable source for searching medical information and tracking the progress in the research field.

Teleconsultants benefit from ChatGPT application as they can receive timely information regarding medical conditions, symptoms, treatment and medication (49). This may help teleconsultants understand patients’ concerns, needs and expectations. In telemedicine, routine tasks include scheduling appointments, sending reminders, and refilling prescriptions. ChatGPT helps explore the virtual patient-physician interaction. A virtual assistant based on ChatGPT may be developed to assistant healthcare professionals in telemedicine to triage patients, and provide remote guidance for home care (65). However, we have observed that participants in such studies may encounter several issues while using the AI application. For example, Caruccio et al. (49) reported that during traditional consultations, patients communicate with physicians who highly prioritize the privacy and confidentiality of their interactions. However, a physician using the ChatGPT application may inadvertently create ambiguity for patients, who have a legitimate right to know whether they are interacting with an AI application or a teleconsultant. Moreover, patients’ privacy and security could potentially be compromised when physicians input sensitive information such as conditions, age, sex, and other factors into the AI system for suggestions. Consequently, significant ethical concerns regarding data privacy, security, accountability, and liability may impose limitations on the utilization of ChatGPT in telemedicine. During the COVID-19 pandemic, ocular oncology faced new challenges with a focus on preventing SARS-CoV-2 exposure. Telemedicine stands out as a major option, setting new standards for ocular treatment during unprecedented times (66). However, a major challenge is to implement the safety measures for healthcare workers and patients to enhance ocular cancer diagnosis and treatment practices.

We noted “unknown” causes for several ocular cancers (25, 27), highlighting areas for future research. Additionally, the ‘Did not reveal’ status under the treatment for choroidal metastasis (28), suggests a lack of consensus or insufficient information regarding treatments, identifying a critical area for further research and development.

## 4 Conclusion

This paper presents an overview of literature detailing the challenges associated with the ocular cancer and explores ways to overcome them using information obtained from the ChatGPT application. This narrative review identified several significant types of ocular cancer, exploring their causes, treatments, and future directions. The strengths and limitations of the ChatGPT application were thoroughly examined in this paper. Furthermore, this review highlighted the challenges associated with ocular cancer. Public health education, telemedicine and remote support through ChatGPT are recommended approaches. Additionally, further research and development in ChatGPT can aid in the prevention of complex eye diseases, such as malignant tumors inside the human eye. Early detection and standardized ocular cancer treatment significantly reduce the risk of developing this disease.

ChatGPT has promising role in managing ocular cancer, offering advantages such as early detection, patient education, treatment plans and research support. However, the successful implementation of ChatGPT in ocular cancer care requires careful addressing of challenges such as data availability, cultural and linguistic sensitivity, regulation and infrastructure support. Addressing these challenges is necessary in future endeavors.

## Author contributions

SA: Conceptualization, Data curation, Formal analysis, Funding acquisition, Investigation, Writing – original draft. AR: Formal analysis, Project administration, Resources, Software, Visualization, Writing – original draft. MH: Methodology, Supervision, Validation, Writing – review & editing.
